# Maize x Teosinte Hybrid Cobs Do Not Prevent Crop Gene Introgression

**DOI:** 10.1007/s12231-012-9195-2

**Published:** 2012-04-26

**Authors:** Nancy B. Chavez, Jose J. Flores, Joseph Martin, Norman C. Ellstrand, Roberto Guadagnuolo, Sylvia Heredia, Shana R. Welles

**Affiliations:** 1 Department of Botany and Plant Sciences, University of California, Riverside, CA 92521-0124 USA; 2 Institut de Botanique, Université of Neuchâtel, 11 rue Emile-Argand, 2009 Neuchâtel, Switzerland

**Keywords:** *Zea mays*, introgression, cob, maize, teosinte, hybridization, dispersal, gene flow, biosafety

## Abstract

**Maize x Teosinte Hybrid Cobs Do Not Prevent Crop Gene Introgression.** Whether introgression from crops to wild relatives can occur is an important component of transgene risk assessment. In the case of maize, which co-occurs with its wild relative teosinte in Mexico, the possibility of introgression has been controversial. Maize is cross-compatible with teosinte, and spontaneous hybridization is known to occur. Some scientists have hypothesized that the maize x teosinte cob infructescence will prevent progeny dispersal, thus preventing introgression. Motivated by a prior study where we found maize x teosinte hybrid fruits naturally dispersed under field conditions, we tested whether hybrid cobs hold their fruits as tightly as maize cobs. We found the force required to detach hybrid fruits was substantially and significantly less than that for maize. Consequently, we expect that introgression of transgenes from maize into teosinte in Mexico should occur largely unimpeded by the hybrid cob.**La mazorca o elote híbrido de maíz x teocintle no impide la introgresión de genes transgénicos provenientes del cultivo.** La introgresión entre el maíz cultivado y el maíz silvestre, o teocintle, es un componente importante en la evaluación ambiental relacionada con los riesgos de la introducción de genes transgénicos. La posibilidad de introgresión entre el maíz domesticado y el teocintle ha sido un tema controversial, en particular en México, donde maíz y teocintle coexisten. El maíz es compatible con el teocintle y la hibridización espontánea ocurre entre ellos. Algunos científicos han planteado como hipótesis que al cruzar el maíz con teocintle, la estructura interna de la infrutescencia que sujeta los frutos conocida como la mazorca de maíz o el elote, impide la dispersión de la progenie evitando que la introgresión ocurra. Los resultados de un estudio previo evidencian la dispersión de los frutos híbridos del maíz x teocintle en condiciones naturales. Motivados por estos resultados, hemos decidido investigar si la mazorca o el elote de las infrutescencias del híbrido sujetan los frutos con una fuerza comparable o mayor a la del maíz. Nuestras mediciones implican que la fuerza necesaria para liberar los frutos híbridos son substancial y significativamente menores que aquellas necesarias para desprender los frutos del maíz. Como conclusión sugerimos que en México, la mazorca o el elote no representan una barrera que impida la introgresión de los genes transgénicos del maíz al teocintle.

## Introduction

When closely related domesticated and wild plants grow within cross-pollination distance, it is not uncommon for hybridization to occur, resulting in movement of crop genes into wild populations (Ellstrand 2003a). If crop alleles successfully establish in wild or weedy populations, such introgression can have significant effects on the recipient population, for example catalyzing the evolution of increased invasiveness (Schierenbeck and Ellstrand 2009). Consequently, crop-to-wild introgression is an important component of risk assessment for genetically engineered crops (Chandler and Dunwell 2008; NRC 2002). Introgression itself is not necessarily a hazard. Whether transgene introgression will have significant consequences will depend on the environment in which it occurs, the biology of the recipient organism, and the biology of the transgene (e.g., Ellstrand and Hoffman 1990; Hokanson et al. 2010; NRC 2002).

For some crops in certain regions, opportunities for transgene flow to wild or weedy populations are nil because no cross-compatible free-living relatives are present. Soybean in North America is an example (Owen 2005). In contrast, transgene flow is nearly certain to occur for certain crops when they grow close to wild relatives, as in the case of sympatric grain sorghum and johnsongrass (Arriola and Ellstrand 1996). But the issue has been controversial for other crops, particularly maize in Mexico where it co-occurs with its wild relatives, various taxa known as “teosintes” (Serratos et al. 1997).

Data supporting spontaneous hybridization between maize (*Zea mays* ssp. *mays*) and what is known as mexicana teosinte (*Z. m.* ssp. *mexicana*) have accumulated over the last century. Wilkes (1967) reported numerous morphological intermediates between maize and mexicana teosinte when he found them growing sympatrically. Experiments conducted by Ellstrand et al. (2007) confirmed that spontaneous hybridization between maize and mexicana teosinte can occur under field conditions, but at extremely low rates, <<1 % per generation. Further, a variety of different descriptive molecular genetic studies, involving almost 100 microsatellite loci (Fukunaga et al. 2005), sequence data from more than two dozen nuclear genes (Ross-Ibarra et al. 2009), and hundreds of SNPs (van Heerwaarden et al. 2011), all revealed that gene flow between maize and teosinte has occurred in the past, but it is unclear how long ago gene flow occurred and whether it continues. Collectively, the previous research demonstrates that hybridization does occur, making contemporary introgression a viable possibility.

Introgression requires more than successful inter-taxon hybridization. The hybrids themselves must be viable and fertile, as well as creating viable, fertile offspring. Common garden field experiments by Guadagnuolo et al. (2006) demonstrated that maize x teosinte hybrids produce more seeds than their wild parent.

Nonetheless, some scientists have proposed that maize x teosinte first generation (F_1_) hybrids might be evolutionary dead ends because those hybrids create an infructescence that is a cob, like its domesticated parent (Martinez-Soriano and Leal-Klevezas 2000). In maize, the cob hampers the fitness of the progeny that it bears (Martinez-Soriano et al. 2002). Typically, maize fruits (botanically, grass family fruits are called “caryopses”) remain firmly attached to the cob (the substantial and persistent rachis of the infructescence). Fruits on a dropped cob remaining in the field will germinate simultaneously. Because the seeds have not dispersed, the seedlings compete intensely with each other so that they die prior to flowering. Thus, attachment to the cob prevents dispersal and is thought to prohibit maize from surviving without human intervention (Martinez-Soriano et al. 2002). In contrast, teosinte fruits disperse readily due to the fact that their thread-like rachis degenerates as their fruits mature. A breeze easily scatters teosinte fruits.

**Fig. 1:**
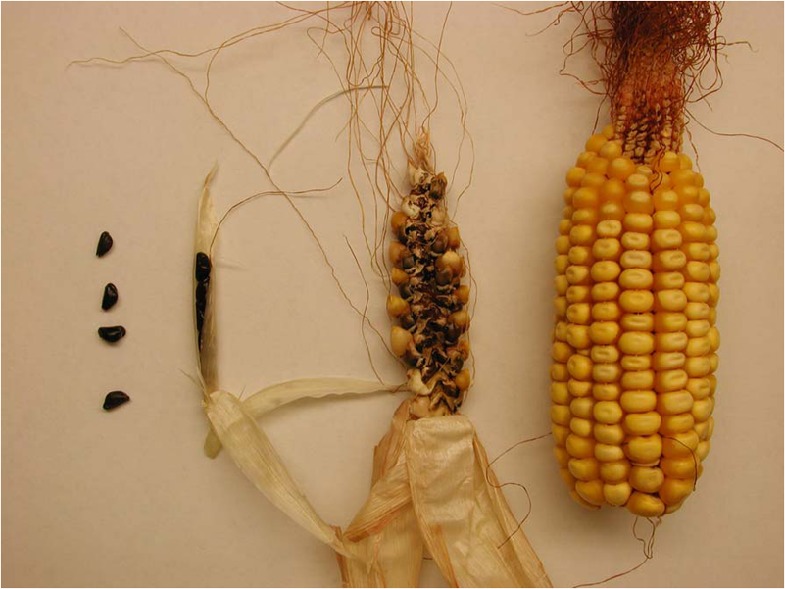
From left to right, teosinte (*Zea mays* ssp. *mexicana*) fruits, teosinte infructescence, F_1_ hybrid infructescence, and maize (*Zea mays* ssp. *mays*) infructescence (photo by Janet Clegg, with permission).

Maize x teosinte hybrids also have a persistent, though reduced, infructescence cob structure (Fig. 1). Although some observers have characterized the hybrid cob as “fragile” (Burbank 1914 as quoted by Wilkes 1967), the opposite view is well established in biosafety discussions about whether engineered maize genes will introgress into teosinte populations. It has been argued that by “not being able to release its seeds” (Martinez-Soriano and Leal-Klevezas 2000), the fruits produced by teosinte x maize hybrids cannot disperse and will suffer the same fate as maize fruits on a cob (e.g., Martinez-Soriano and Leal-Klevezas 2000; Martinez-Soriano et al. 2002). According to this argument, introgression of maize alleles into teosinte should be nil or severely hampered. This hypothesis is so prevalent that regulatory decisions regarding the deregulation of transgenic maize often depend on it. For example, the recent USDA–APHIS (United States Department of Agriculture–Animal and Plant Health Inspection Services) (2009) decision document for “alpha-amylase event 3272 corn” mentions lack of seed dispersal from hybrids three different times as a “constraint on introgression” from the crop to the wild relative. However, that hypothesis has never been tested; the relative ease of fruit shattering from maize x teosinte hybrids has never been quantified.

Observations made during a prior field study involving maize, teosinte, and their hybrids (Guadagnuolo et al. 2006) motivated our current study. Specifically, dispersed individual hybrid fruits were observed underneath parental plants (R. Guadagnuolo, pers. obs.). The fruits were collected and confirmed to be produced by hybrid plants because they were partly covered with glumes, in contrast to fully-enclosed teosinte fruit and unenclosed maize fruit. The field was carefully inspected for dispersed fruit. A total of more than 300 hybrid fruits dispersed from 167 hybrid plants were found loosely scattered in the experimental plot. In addition, more than 1,200 fruits were found in the plot that had been set by the 196 experimental teosinte plants. Not a single fruit was found to be dispersed from the 400 maize plants in the experiment.

These findings stimulated us to compare quantitatively the potential for maize, teosinte, and F_1_ hybrid dispersal in order to determine the extent to which cob structure is an absolute barrier to fruit dispersal, and consequently, introgression.

## Materials and Methods

### Plant Material

We compared three types of infructescences from (1) teosinte, *Zea mays* ssp. *mexicana,* (2) maize, *Zea mays* ssp. *mays*, and (3) their F_1_ hybrids. As part of a prior study at the University of California Riverside Agricultural Experiment Station in Riverside, California (Guadagnuolo et al. 2006), teosinte infructescences were harvested from plants grown from seeds descended from a 1972 collection by George Beadle (provided by Professor J. Giles Waines) and subsequently dried. Hybrid infructescences were also harvested from plants grown in that prior study. Those hybrid plants were the result of hand-crosses between the teosintes and commercial field maize (for details see Guadagnuolo et al. 2006). The dry maize infructescences (ears) we used for this study were obtained from the Carolina Biological Supply® company.

### Measurement of Force

We measured the force needed to free the fruits using a Chatillon® DEM 50 force gauge. Modified fish hooks were attached to the device for the purpose of pulling individual fruits from the cobs. The device was set to peak tension; the force was measured in Newtons (N).

Fruits were pulled from the distal, middle, and proximal portions of each infructescence; 18 teosinte individuals, 54 hybrid individuals, and 24 maize individuals were used. Each maize and hybrid infructescence was fastened horizontally by a table clamp to hold it firmly. In the case of teosinte, fruits are attached by such a fragile rachis that a horizontal position caused multiple fruits to be dislodged spontaneously. Thus, teosinte infructescences were clamped in place vertically, and grains were dislodged using horizontal force. Because the fruits of hybrids and maize are densely attached, fruits adjacent to and above the selected grain were carefully removed to make room for the hook.

Fishhooks of varying sizes were used to best accommodate varying fruit sizes. The fishhook was affixed to the individual fruit and pulled with a forward motion until the fruit detached. The reading for the peak tension was then recorded. All trials were performed by the same person to minimize variation.

### Statistical Analysis

Given that our data could not be normalized, we used a non-parametric Kruskal-Wallis test with two degrees of freedom to test for differences between the forces necessary to dislodge fruits from our three different samples. This test was followed by a pairwise comparison between the data of the maize, teosinte, and hybrids using a Wilcoxon rank sum test with a Bonferonni adjustment for multiple comparisons so that specific significant differences could be elucidated. The same tests were done to detect differences in dispensability of different portions of the infructescence. Calculations were done using R statistical package (R Core Development Team 2004).

## Results

**Fig. 2:**
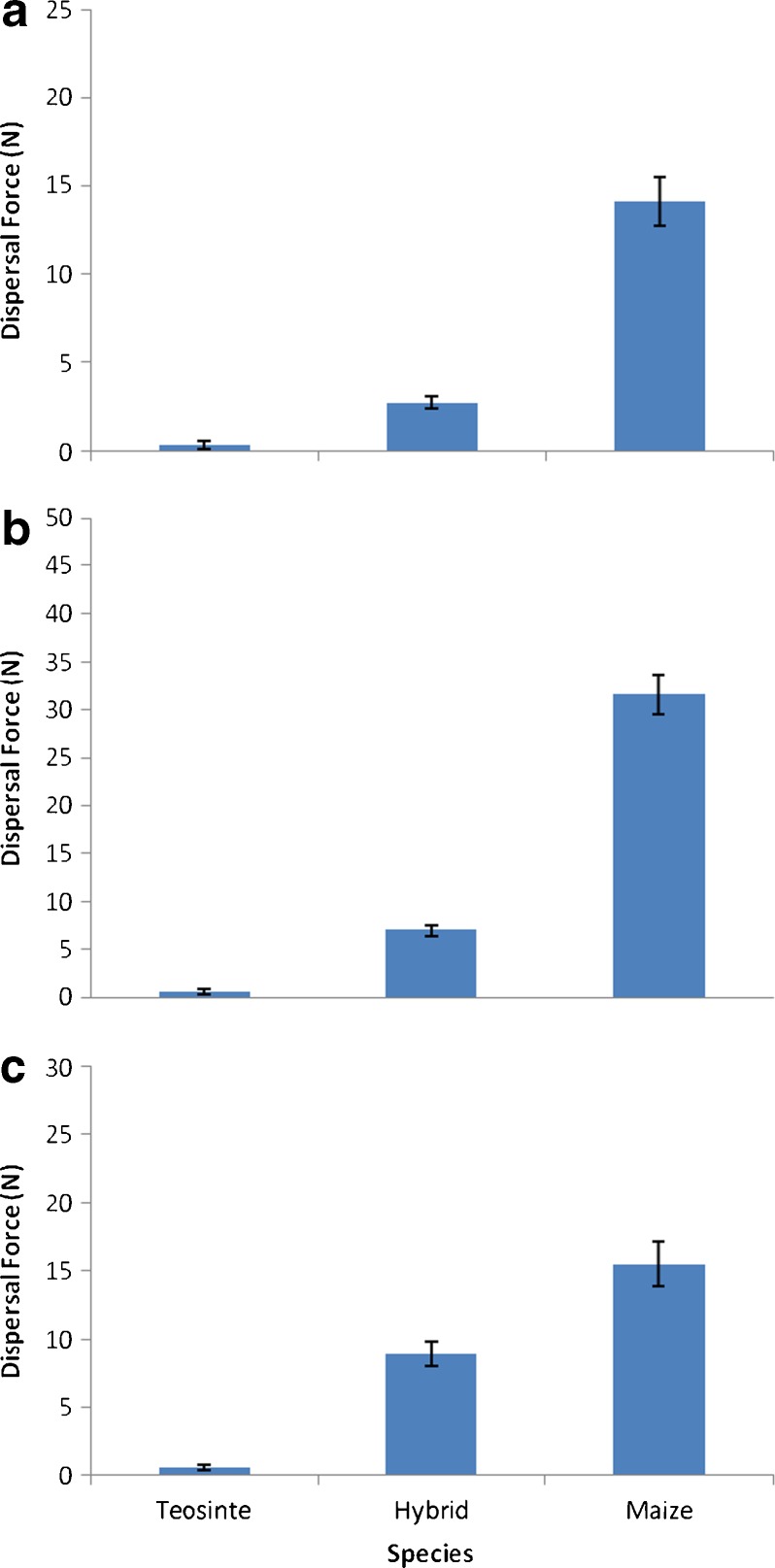
Mean dispersal force (± SEM) of caryopses from maize, teosinte, and hybrid infructescences where (**a**) is from distal section, (**b**) from mid-section, and (**c**) from proximal section of each species. Pairwise comparisons between each species at every location showed significant differences between species based on results from a pairwise Wilcoxon test employing a Bonferroni correction for multiple comparisons (nb all p-values smaller than 1x10-7). Proximal caryopses dislodged with significantly less force than mid or distal sections.

For all three positions measured on the infructescence, we found substantial differences in the force necessary to detach the fruits of teosinte, maize, and their hybrid (Fig. 2). A Kruskal-Wallis test comparing the detachment force for teosinte, hybrids, and maize showed significant heterogeneity with a P value <6.3 x 10^-16^. For each position, teosinte fruits required the least force (x = 0.34 N for distal portion of infructescence, x = 0.55 N for the middle, and x = 0.56 N for the proximal). Maize fruits required the most force (x = 13.88 N for distal portion of infructescence, x = 33.3 N for the middle, and x = 15.9 N for the proximal). Hybrid fruits required intermediate force (x = 2.77 N for distal portion of infructescence, x = 6.95 N for the middle, and x = 8.88 N for the proximal). Pairwise comparisons using Wilcoxon Rank Sum test with a Bonferroni correction for multiple comparisons demonstrated highly significant differences between the average shattering forces for each pair of comparisons at each of the positions (all p-values were less than 1 x 10^-7^).

We also found overall systematic and significant differences in shattering forces between the different infructescence positions. A Kruskal-Wallis test comparing the detachment force for distal, mid, and proximal positions showed significant heterogeneity with a P = 1.40 x 10^-4^. Pairwise comparisons using Wilcoxon Rank Sum test with a Bonferroni correction for multiple comparisons showed no significant difference between the proximal and middle positions, but found the average shattering force for the distal position was significantly lower than both the proximal and middle positions (P = .00074 and P = .00086, respectively).

## Discussion

We found significant differences in the force necessary to detach fruits from teosinte, maize, and their hybrids. Not surprisingly, we found teosinte fruits are easily dispersed. In contrast, depending on the position on the cob, the force necessary to detach maize fruits was more than 50-fold greater than that of teosinte. We found that the hybrids require an intermediate force for detachment, considerably more than teosinte and considerably less than maize. These results contrast with prior claims that maize x teosinte F_1_ hybrid cobs should have the same dispersability as maize cobs (Martinez-Soriano and Leal-Klevezas 2000; Martinez-Soriano et al. 2002).

Our results suggest that the potential for dispersal, and consequently introgression, exists. They are congruent with our prior discovery of dispersed hybrid fruits that we mentioned in the Introduction. They are compatible with our observation that stepping on a hybrid cob typically results in the release of fruits, while stepping on a maize cob typically has no effect. There is no question that hybrids disseminate more efficiently than maize, but less efficiently than teosinte. It is clearly wrong to assume that the F_1_ generation hybrid cob is an absolute barrier to subsequent introgression. After all, hybrid cobs would be expected to occur in agro-ecosystems where cobs could be broken into individual fruits or small fragments by a plow, foot, or hoof; units would be small enough to be easily and passively dispersed. Given the finding of significant variation in force required for dispersal between different portions of the infructescence, we would expect that fruits from the distal portion would be more easily dispersed.

Another avenue of introgression has been overlooked. Even if the hybrid plants were 100 % seed-sterile, under field conditions they can serve as male parents and backcross to nearby pure teosinte plants because their flowering time overlaps substantially (Guadagnuolo et al. 2006). We have preliminary evidence that such back-crossing can occur in the field (Guadagnuolo, pers. obs.).

The foregoing data, our experimental results, and our prior observations of dispersed hybrid seed, as well as prior genetic data of others supporting maize-to-teosinte introgression (Blancas et al. 2002; Fukunaga et al. 2005; Ross-Ibarra et al. 2009; Wilkes 1967), demonstrate that introgression of maize alleles into teosinte populations is not necessarily hampered by the hybrid cob. Introgression may not occur at high rates, but there is every reason to believe that maize-to-teosinte introgression may occur wherever mexicana teosinte and maize are within cross-pollinating distance. Furthermore, minimal gene flow can have a substantial evolutionary effect; population genetics theory demonstrates that for neutral alleles a single successful immigrant every other generation is sufficient to allow introgression to proceed (Wright 1969).

Such introgression has immediate consequences for transgenic maize biosafety. It should be assumed that if transgenic maize is grown spatially close enough to teosinte to hybridize, then some level of hybridization is apt to occur if its flowering time overlaps with that of teosinte. The resulting hybrid progeny will have opportunities to continue to the next steps to introgression either by mating among themselves and creating F_2_ recombinants or by fathering seeds on nearby teosinte plants to create BC_1_s. Transgene flow in itself does not necessarily represent a hazard (NRC 2002). But if there is a reason to judge that the specific transgene in question will create a significant hazard in the introgressed descendants, such exposure should be prevented. We recognize that a hazard might be identified as such not only from natural science considerations, but also from those of social science (NRC 1996).

Teosinte populations could serve as a genetic bridge for transgenes to move from one maize population to the next. Once a transgene has made its way into a teosinte population, unless it confers a fitness disadvantage, it is likely to persist in the wild population, outcrossing to all maize populations within cross-pollination distance (Ellstrand 2003a).

That bridge could occur over time as well as over space. If an introgressed teosinte population persists over generations, the potential exists for that population to outcross with a future nearby maize plantation, reintroducing the transgene locally. In the regions of Mexico where teosinte is a part of the native flora, maize is often grown as landraces, where seed is saved every year and replanted (Ellstrand 2003b). Thus, the transgene could have an opportunity to evolve undetected. Our data suggest that biosafety regulators in regions where teosinte occurs should not only consider the impact of a transgene on teosinte populations, as they already do, but also the potential for the impacts of long-term persistence of the transgene in teosinte populations with the likelihood of those populations acting as a genetic bridge back to maize.
